# Ontario Veterinary College

**Published:** 1896-05

**Authors:** 


### ONTARIO VETERINARY COLLEGE.

The closing exercises were held in the college building, Toronto, on Saturday, March 28th, and were presided over by Prof. Andrew Smith. Among those present with him on the platform were Lieutenant-Governor Kirkpatrick, Robert Jaffray, John Aikins, R. J. Score, Dr. Duncan, Dr. Sweetapple, W. W. Withrow, Dr. J. O. Orr, and others.

Professor Smith addressed the students, calling attention to the fair attendance at the college this year despite the depression, also referring to the fact that the veterinary profession was improving at the present time.

Dr. Duncan then read the list of graduates and prize-winners.

*Graduates:* William Agnew, Belgrave, Ont.; Fred. A. Armstrong, Fergus, Ont.; T. H. Agnew, Evanston, Ill.; Simon Beattie, Waterloo, Wis.; R. J. Besteel, Grafton, North Dakota; John F. J. Black, Dublin, Ireland; Paul Dudley Bray, Cannington Manor, Assa.; Arthur Brechter, Beaver Dam, Wis.; William G. Bunnell, Heywood, Manchester, Eng.; Basil Aubin Brown, Highbury, London, Eng.; Francis J. Braund, McGregor, Man.; Patrick Cain, New Perth, P. E. I.; Merton J. Chrisman, Frewsburg, N. Y.; John L Clark, Stratford, Ont.; Keller Lark, Cooperstown, N. Y.; Edgar W. Culley, Avon, N. Y.; Herbert Caldwell, Louisville, Ky.; John Cramer, Elwood, Ind.; Robert H. Craig, Bushton, Ill.; John A. Campbell, Teeswater, Ont.; Edward Dingman, Maplewood, Ont.; Richard A. Dennis, Adams, N. Y.; John R. Donahue, St. Thomas, Ont.; Charles H. Drake, New Haven, Conn.; F. J. De Land, Clinton, N. Y.; A. F. Elliott, Grandin, North Dakota; Henry T. Ferguson, Lake Geneva, Wis.; John B. Finch, Ramseys, New Jersey; Albert B. Gay, Randolph, Vt.; D. F. George, Overton, O.; John F. Gillespie, Ridge Farm, Ill.; William J. Grady, Quincy, North Dakota; N. O. Gilbert, Brooklyn, N. Y.; A. Gibson, Berlin, Ont.; Leopold Hay, Warsaw, Poland; Charles Haas, St. George, Ont.; William W. Hall, Leeds, P. Q.; Albert B. Hecker, Albany, N. Y,; J. B. Hill, Canandaigua, N. Y.; Roy F. Hoadley, Yorkville, Ill.; Walter E. Howe, Delphi, N. Y.; Walter N. J. Hurt, Matlock, Bath, Eng.; Alfred P. Husband, Nassagaweya, Ont.; T. M. Hadwen, Wheatland, North Dakota; George R. Inglis, Campbellville, Ont.; Johnson J. Irwin, Sheffordvale, Que.; Roy E. Jackson, Tyler Hill, Pa.; P. H. Johnson, Racine, Wis.; J. E. Johnson, Texas, O.; Elmer H. Judkins, Lake Mohawk, N. Y.; Chester P. Keith, Bridgewater, Mass.; Thomas Le Roy Kelly, Lakeside, O.; John C. Kingan, Pittsburg, Pa.; Rudolph Kjerner, Rochester, Minn.; William H. Kenwell, Cuba, N. Y.; John Lawrason, St. George, Ont.; Harry L. Leberman, Meadville, Pa.; William S. Longacre, Mantz, Pa.; J. T. Leary, Ellicottville, N. Y.; Newton G. Le Gear, Imlay City, Mich.; John Dennis Macdonald, Jr., London, Eng.; Robert W. McDonald, Jackson, Mich.; Alexander J. McKay, Tavistock, Ont.; Peter McNeal, Shickshinny, Pa.; C. H. McGillicuddy, Lewiston, Me.; Daniel Maher, Godfrey, Ill.; H. E. Marshall, Ottawa, Ont.; G. R. C. Merriam, Negril, Jamaica, W. Indies; Sheard Moore, Lowell, Mass.; Lemuel Mulligan, Ottawa, Ont.; W. N. Neil, Tower Hill, Ill.; Brittain E. Nevel, Huntersville, Pa.; Lewis A. Nutting, Syracuse, N. Y.; John Oliver, Jr., Columbus, Miss.; J. D. Paxton, St. George, Ont.; H. C. Peters, Greenleaf, Minn.; J. Orville Reed, Danville, Pa.; Andrew W. Reilly, Grand Valley, Ont.; Llewellyn T. Richards, Granville, O.; James L. Riddell, Denver, Col.; Peter A. Robinson, Emerson, Man.; Louis D. Regan, Emporia, Kan.; Herman R. Ryder, Delphi, N. Y.; F. G. Scammel, Lafayette, N. Y.; Charles A. Seaton, Bloomington, Ont.; S. L. Shaw, Summer Hill, Ill.; Geo. F. Sheppard, Edinburgh, North Dakota; Thomas Veitch Simpson, Yorkton, N. W. T.; John J. Sparrow, Victoria, B. C.; Ray J. Stancliff, Derby, N. Y.; Wesley C. Stephens, Newmarket, Ont.; Donald A. Stewart, Ivan, Ont.; John H. Summers, Oswego, N. Y.; Zera Strong, Carleton Place, Ont.; James A. Stevenson, Morden, Man.; Francis C. Simpson, Bridlington Quay, Eng.; J. W. Tooley, Lansing, Ont.; J. Turnbull, Lowville, Ont.; J. H. Ellsworth Vrooman, Vroomanton, Ont.; Richard E. Willis, Brampton, Ont.; Arthur R. Walsh, Buffalo, N. Y.; William Walker, Kingston, Ont.; M. A. Whinster, Portage la Prairie, Man.; E. J. Will, Moore's Store, Va.; Arthur Edward Williamson, Queenstown, Ireland; David B. Wilson, London, Ont.; George E. Wilson, Carleton Place, Ont.; F. Lockwood-Wingate, Kingston, Jamaica, W. I.; R. W. Wilkinson, Drumbo, Ont.; A. C. Woods, Council Bluffs, Iowa; A. G. Young, Bristol, Quebec.

*Seniors by Departments:* Pathology (seniors): silver medal, 1st prize, H. R. Ryder; 2d prize, F. Lockwood-Wingate; 3d prize, R. J. Stanclift, P. D. Bray. Honors: F. A. Armstrong, J. F. J. Black, J. L. Clark, E. H. Cully, H. Caldwell, R. A. Dennis, J. B. Finch, H. T. Ferguson, L. Hay, W. N. J. Hurt, W. E. Howe, G. R. Inglis, J. E. Johnson, J. Johnson, R. Kjerner, C. P. Keith, G. R. C. Merriam, A. J. McKay, W. N. Neil, L. A. Nutting, J. D. Paxton, J. L. Riddell, L. T. Richards, H. C. Peters, P. A. Robinson, T. V. Simpson, F. C. Simpson, C. A. Seaton, D. A. Stewart, J. Turnbull, J. H. E. Vrooman, A. R. Welsh, G. E. Wilson, E. J. Will, A. G. Young.

Materia Medica (seniors): 1st prize, J. F. J. Black; 2d prize, H. F. Ferguson and F. L. Wingate (equal); 3d prize, C. P. Keith. Honors: P. D. Bray, J. P. Fitzgerald, H. E. Marshall, G. R. C. Merriam, R. J. Stanclift, F. C. Simpson, T. V. Simpson.

Chemistry (seniors): 1st prize, F. C. Simpson; 2d prize, R. J. Stanclift; 3d prize, B. A. Brown. Honors: J. F. J. Black, R. H. Craig, E. M. Culley, J. B. Finch, J. P. Fitzgerald, J. E. Johnson, C. P. Keith, G. R. C. Merriam, L. D. Ryan, H. R. Ryder, D. A. Stewart.

Morbid Anatomy (seniors): Prize, H. R. Ryder. Honors: P. D. Bray, R. J. Stanclift, G. R. C. Merriam, J. R. Donohue, W. E. Howe, J. F. J. Black, F. L. Wingate.

Anatomy (seniors): 1st prize, silver medal, R. J. Stanclift; 2d prize, J. F. J. Black; 3d prize, E. W. Culley. Honors: Wm. Agnew, P. D. Bray, B. A. Brown, W. G. Bunnell, R H. Craig, J. L. Clark, M. J. Chrisman, Herbert Caldwell, J. R. Donahue, R. A. Dinnis, T. H. Ferguson, W. E. Howe, W. W. J. Hurt, C. A. Haas, W. W. Hall, J. B. Hill, L. Hay, G. R. Inglis, J. E. Johnston, E. H. Judkins, R. E. Jackson, A. Kjerner, C. P. Keith, W. S. Longacre, G. R. C. Merriam, A. J. McKay, J. Oliver, J. D. Paxton, H. R. Ryder, P. A. Robinson, T. V. Simpson, F. C. Simpson, S. L. Shaw, J. J. Sparrow, G. T. Sheppard, D. A. Stewart, J. Turnbull, A. R. Walsh, E. J. Will, R. E. Willis, F. L. Wingate, A. G. Young.

Dissected Specimens (seniors): Gold medal, given by Toronto Industrial Exhibition Association, J. F. J. Black; 2d prize, $30, F. C. Simpson.

Eutozoa (seniors): P. D. Bray. Honors: W. Agnew, S. Beattie, J. F. J. Black, J. A. Campbell, R. H. Craig, E. W. Culley, L. Hay, W. E. Howe, G. R. C. Merriam, J. Oliver, J. D. Paxton, S. L. Richards, H. R. Ryder, T. V. Simpson, R. J. Stanclift, S. L. Shaw, F. C. Simpson, D. A. Stewart, F. L. Wingate, A. C. Woods.

Physiology (seniors): 1st prize, silver medal, P. D. Bray; 2d prize, G. R. C. Merriam; 3d prize, T. V. Simpson. Honors: J. F. J. Black, B. A. Brown, R. H. Craig, E. W. Culley, J. R. Donahue, J. B. Finch, L. Hay, W. E. Howe, G. R. Inglis, J. D. Macdonald, Wm. Neill, H. R. Ryder, S. L. Shaw, R J. Stanclift, J. Turnbull, A. C. Woods, F. L. Wingate.

Best general examination: G. R. C. Merriam, gold medal given by the Ontario Veterinary Medical Association. Honors: J. F. J. Black, T. H. Ferguson, Leopold Hay, W. N. Neil, L. T. Richards, H. R. Ryder.

*Juniors by Departments*. Pathology (juniors): 1st prize, W. H. Wheeler; 2d prize, G. E. Totten; 3d prize, W. J. Meloche and E. L. Bertram (equal). Honors: H. Buss, F. W. Bryant, G. C. Bowen, W. Brennen, J. B. Campbell, W. Caister, G. R. Caldwell, R. C. Cliff, T. J. Cooper, C. W. Clark, J. Dodd, G. P. Hayter, G. Hilton, F. Hodgson, J. Johnston, J. J. Keliher, F. A. Messinger, A. A. McLachlan, J. A. McKinnon, W. O. McHugh, G. A. McKenzie, I. S. Norton, R. D. Orr, T. M. Owen, J. H. Pierce, H. Pegan. J. R. Rochester, W. W. Richards, G. Rowencroft, J. Miller, W. McElroy, R. Marshall.

Anatomy (juniors): 1st prize, silver medal, G. E. Totten; 2d prize, R. D. Orr; 3d prize, R. C. Cliff and G. R. Caldwell (equal). Honors: Henry Buss, J. B. Campbell, Thomas Cooper, W. Caistor, George Hilton, Fred. Hodgson, J. N. Johnston, J. J. Keliher, A. McLachlan, W. A. McHugh, W. F. McIlroy, W. J. Meloche, J. P. Miller, I. S. Norton, W. W. Richards, G. V. Rowcroft, G. P. Statter, J. W. Will, S. A. H. Winsloe.

Physiology (juniors): 1st prize, silver medal, R. C. Cliff; 2d prize, G. Hilton; 3d prize, W. O. McHugh. Honors: C. W. Clark, J. J. Keliher, J. P. Miller, W. J. Meloche, W. W. Richards, G. V. Rowcroft, W. H. Wheeler.

Chemistry (juniors): 1st prize, George Hilton; 2d prize, G. V. Rowcroft.

Histology and Microscopy (juniors): 1st prize, T. J. Cooper; 2d prize, C. W. Clark; 3d prize, R. Cliff. Honors: E. Bertram, C. H. Bugbee, F. W. Bryant, T. W. Brown, G. R. Caldwell, J. B. Campbell, H. Chaney, J. Dodd, W. H. Erwin, G. J. Grange, G. R. Hayter, R. C. Harris, G. Hilton, F. Hodgson, J. N. Johnston, J. J. Keliher, G. A. McKenzie. W. F. McElroy, Wm. McHugh, J. A. McKinnon, A. A. McLachlan, R. Marshall, W. J. Meloche, F. G. Messinger, J. S. Norton, R. D. Orr, T. Owen, J. H. Pierce, H. Pegan, W. W. Richards, J. R. F. Rochester, G. V. Rowcroft, G. Statter, A. E. Tweedle, C. A. Taylor, J. A. Winsloe.

Primary Examinations. Anatomy: F. G. Atwood, A. McK. Brock, A. E. Dennis, Joseph Gregg, R. J. Marion. W. D. Monk, W. H. Orme.

Materia Medica: J. P. Fitzgerald, H. F. Hartnett, G. H. Munroe, W. H. Orme, G. A. Wehr.

After the presenting of the prizes by Lieutenant-Governor Kirkpatrick, he gave a short and congratulatory address to the students, paying a warm compliment to the college on the instruction imparted. He believed in the future skilled veterinary surgeons would be more in demand than ever. That the necessity for a thorough and scientific inspection of all dairies and abattoirs was becoming recognized, therefore a new field was being opened for employment.

Several other gentlemen gave short and interesting addresses.

At the close of the proceedings Mr. J. E. Johnson, president of the graduating class, came forward, and, after reading an address to Professor Smith, presented him with a magnificent picture of the class. Professor Smith in a short reply expressed his thanks to the students for this expression of their good feeling toward himself and the college.

